# Saving Mothers, Giving Life: It Takes a System to Save a Mother

**DOI:** 10.9745/GHSP-D-18-00427

**Published:** 2019-03-11

**Authors:** Claudia Morrissey Conlon, Florina Serbanescu, Lawrence Marum, Jessica Healey, Jonathan LaBrecque, Reeti Hobson, Marta Levitt, Adeodata Kekitiinwa, Brenda Picho, Fatma Soud, Lauren Spigel, Mona Steffen, Jorge Velasco, Robert Cohen, William Weiss

**Affiliations:** a Bureau for Global Health, U.S. Agency for International Development, Washington, DC, USA.; b Division of Reproductive Health, U.S. Centers for Disease Control and Prevention, Atlanta, GA, USA.; c Centers for Disease Control and Prevention, Lusaka, Zambia. Now retired.; d U.S. Agency for International Development, Lusaka, Zambia. Now based in Monrovia, Liberia.; e Bureau for Global Health, U.S. Agency for International Development, Washington, DC. Now with ICF, Rockville, MD, USA.; f Bureau for Global Health, U.S. Agency for International Development and RTI, Washington, DC, USA. Now with Palladium, Abuja, Nigeria.; g Baylor College of Medicine Children's Foundation-Uganda, Kampala, Uganda.; h Infectious Diseases Institute, College of Health Sciences, Makerere University, Kampala, Uganda.; i Centers for Disease Control and Prevention, Lusaka, Zambia. Now an independent consultant, Gainesville, FL, USA.; j ICF, Fairfax, VA, USA. Now with Ariadne Labs, Boston, MA, USA.; k U.S. Agency for International Development, Papua, New Guinea.

## Abstract

**Background:**

Ending preventable maternal and newborn deaths remains a global health imperative under United Nations Sustainable Development Goal targets 3.1 and 3.2. Saving Mothers, Giving Life (SMGL) was designed in 2011 within the Global Health Initiative as a public–private partnership between the U.S. government, Merck for Mothers, Every Mother Counts, the American College of Obstetricians and Gynecologists, the government of Norway, and Project C.U.R.E. SMGL's initial aim was to dramatically reduce maternal mortality in low-resource, high-burden sub-Saharan African countries. SMGL used a district health systems strengthening approach combining both supply- and demand-side interventions to address the 3 key delays to accessing effective maternity care in a timely manner: delays in seeking, reaching, and receiving quality obstetric services.

**Implementation:**

The SMGL approach was piloted from June 2012 to December 2013 in 8 rural districts (4 each) in Uganda and Zambia with high levels of maternal deaths. Over the next 4 years, SMGL expanded to a total of 13 districts in Uganda and 18 in Zambia. SMGL built on existing host government and private maternal and child health platforms, and was aligned with and guided by Ugandan and Zambian maternal and newborn health policies and programs. A 35% reduction in the maternal mortality ratio (MMR) was achieved in SMGL-designated facilities in both countries during the first 12 months of implementation.

**Results:**

Maternal health outcomes achieved after 5 years of implementation in the SMGL-designated pilot districts were substantial: a 44% reduction in both facility and districtwide MMR in Uganda, and a 38% decrease in facility and a 41% decline in districtwide MMR in Zambia. Facility deliveries increased by 47% (from 46% to 67%) in Uganda and by 44% (from 62% to 90%) in Zambia. Cesarean delivery rates also increased: by 71% in Uganda (from 5.3% to 9.0%) and by 79% in Zambia (from 2.7% to 4.8%). The average annual rate of reduction for maternal deaths in the SMGL-supported districts exceeded that found countrywide: 11.5% versus 3.5% in Uganda and 10.5% versus 2.8% in Zambia. The changes in stillbirth rates were significant (−13% in Uganda and −36% in Zambia) but those for pre-discharge neonatal mortality rates were not significant in either Uganda or Zambia.

**Conclusion:**

A district health systems strengthening approach to addressing the 3 delays to accessing timely, appropriate, high-quality care for pregnant women can save women's lives from preventable causes and reduce stillbirths. The approach appears not to significantly impact pre-discharge neonatal mortality.

## Abstract (precis)

A multi-partner effort in Uganda and Zambia employed a districtwide health systems strengthening approach, with supply- and demand-side interventions, to address timely use of appropriate, quality maternity care. Between 2012 and 2016, maternal mortality declined by approximately 40% in both partnership-supported facilities and districts in each country. This experience has useful lessons for other low-resource settings.

## INTRODUCTION

Despite a 45% drop in global maternal deaths between 1990 and 2015,^1^ maternal mortality remains an intractable public health problem in many low-resource settings. Only 1 sub-Saharan African country, Rwanda, achieved the target for Millennium Development Goal 5 (reduce by three-quarters, between 1990 and 2015, the maternal mortality ratio).^1^^,^^2^ Attempts have been made to bring high-level visibility to the cause, but many countries have not directed sustained political attention or sufficient resources to eliminate preventable maternal mortality^3^—despite solid evidence of the profound effects a mother's death has on her family, her community, and on development in general.^4^^,^^5^ The situation is particularly dire in sub-Saharan African countries where 60% of global maternal deaths occur.^1^^,^^5^^,^^6^ In these countries, obstetrical risk is compounded by high fertility rates, raising the lifetime risk of death due to childbirth to 1 in 36, compared with 1 in 8,400 in the European Union.^7^^–^^9^

Newborns fare no better. Globally, the reduction in newborn deaths has not kept pace with the reduction of deaths in children under age 5, with newborn deaths now contributing to nearly half of child mortality.^1^ The average neonatal mortality rate is 27 deaths per 1,000 live births in low-income countries compared with 3 deaths per 1,000 live births in high-income countries. Eight of the 10 most dangerous places to be born are in sub-Saharan Africa.^10^

In 2011 the Office of the Global Health Initiative (GHI) within the U.S. Department of State was tasked with designing an endeavor that would bring public and private investment together with committed Ministry of Health (MOH), national, and district leaders to address maternal mortality in sub-Saharan Africa.^11^^,^^12^ It was felt that a highly visible, well-financed, bold initiative similar to the U.S. President's Emergency Plan for AIDS Relief (PEPFAR), the President's Malaria Initiative, and Feed the Future was needed to inspire and recruit new public and private actors to the cause, while energizing and mobilizing the global health and development communities. The resulting initiative was Saving Mothers, Giving Life (SMGL), a public–private partnership. SMGL was composed of 6 U.S. agencies: GHI; the United States Agency for International Development (USAID) (which took over oversight of the partnership from GHI in July 2012 and responsibility as Secretariat from Merck for Mothers in 2014); the U.S. Centers for Disease Control and Prevention (CDC); the Office of the Global AIDS Coordinator (OGAC); Peace Corps; and the Department of Defense. It also included the Governments of Norway (became inactive in 2014), Uganda, Zambia, and Nigeria (joining in 2015 as the third SMGL country and slated to end in October 2019); Merck for Mothers; Every Mother Counts; the American College of Obstetricians and Gynecologists; and Project C.U.R.E (joined the partnership in 2013). SMGL's initial goal was to decrease maternal mortality by 50% in 1 year in SMGL-designated districts in Uganda and Zambia, building on existing national public health platforms and systems, and aligning with country maternal health strategies and aspirations.^13^^,^^14^ At the end of the first phase of the partnership, the time frame for the goal was extended to the close of the initiative in 2017. An additional goal of reducing the neonatal mortality rate by 30% was added in 2013.

The Saving Mothers, Giving Life journal supplement consists of 11 articles on the SMGL initiative. The articles describe the formation and function of the partnership, the SMGL theory of change, programming approach and costs, and the results achieved in Uganda and Zambia where implementation ended in October 2017 (Table 1). It aims to answer key questions about the initiative and identify outstanding implementation issues. Results from Nigeria will be reported in 2019 after implementation in that country has ended.

**TABLE 1.** Saving Mothers, Giving Life Supplement Articles

Article No.	Article Title
1	Saving Mothers, Giving Life: it takes a system to save a mother
2	Impact of the Saving Mothers, Giving Life approach on decreasing maternal and perinatal deaths in Uganda and Zambia
3	Addressing the first delay in Saving Mothers, Giving Life districts in Uganda and Zambia: approaches and results for increasing demand for facility delivery services
4	Addressing the second delay in Saving Mothers, Giving Life districts in Uganda and Zambia: reaching appropriate maternal care in a timely manner
5	Addressing the third delay in Saving Mothers, Giving Life districts in Uganda and Zambia: ensuring adequate and appropriate facility-based maternal and perinatal health care
6	The costs and cost-effectiveness of a district-strengthening strategy to mitigate the 3 delays to quality maternal health care: results from Uganda and Zambia
7	Saving lives together: a qualitative evaluation of the Saving Mothers, Giving Life public-private partnership
8	Community perceptions of a 3-delays model intervention: a qualitative evaluation of Saving Mothers, Giving Life in Zambia
9	Did the Saving Mothers, Giving Life initiative expand timely access to lifesaving care in Uganda? A spatial district-level analysis of travel time to emergency obstetric and newborn care
10	Saving Mothers, Giving Life approach for strengthening health systems to reduce maternal and newborn deaths in 7 scale-up districts in northern Uganda
11	Sustainability and scale of the Saving Mothers, Giving Life approach in Uganda and Zambia

SMGL's initial goal was to decrease maternal mortality by 50% in 1 year in selected districts in Uganda and Zambia.

## THEORY OF CHANGE

**FIGURE 1:**
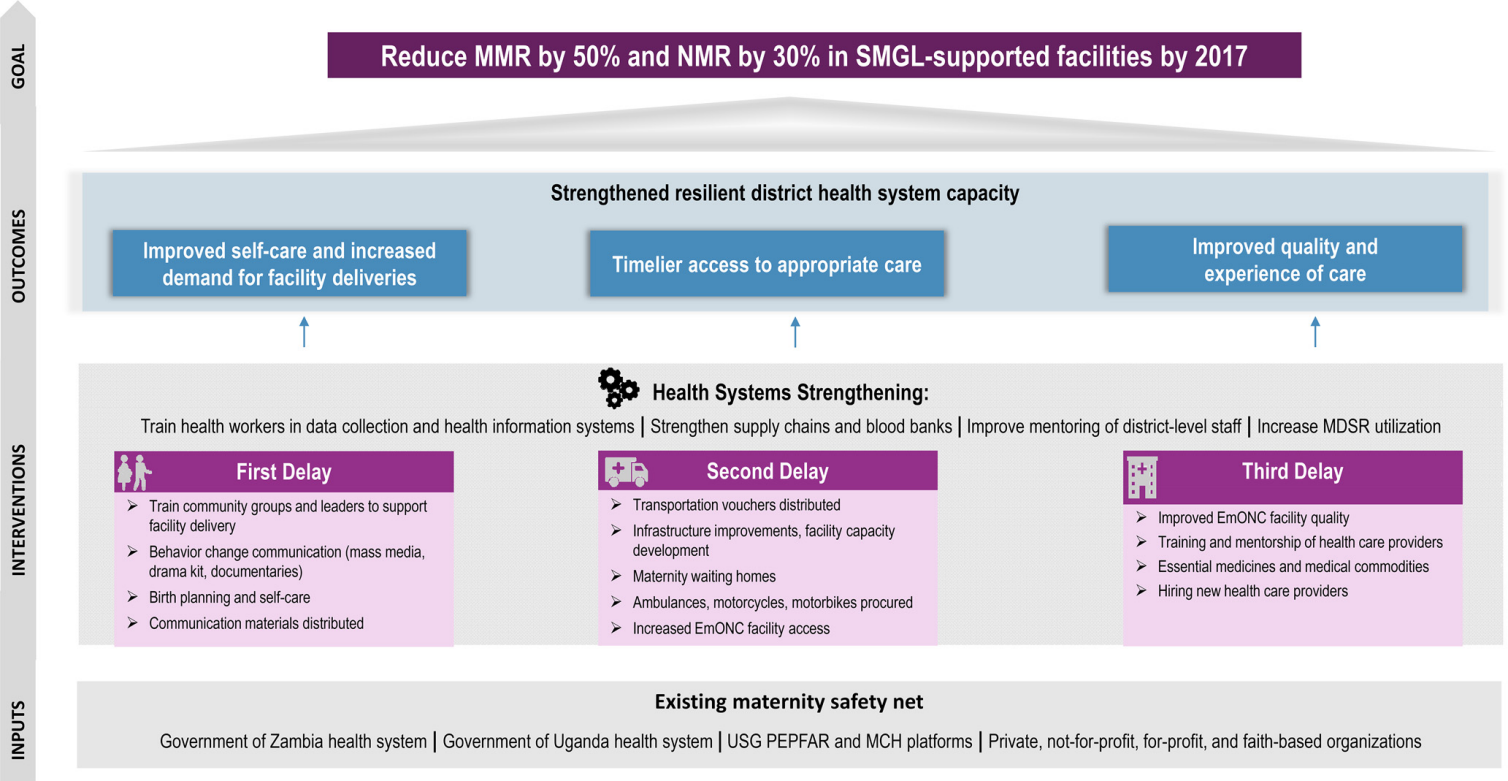
Saving Mothers, Giving Life Theory of Change Model Abbreviations: EmONC, emergency obstetric and newborn care; MCH, maternal and child health; MPDSR, maternal and perinatal death surveillance and response; MMR, maternal mortality ratio; NMR, neonatal mortality rate; PEPFAR, U.S. President's Emergency Plan for AIDS Relief; SMGL, Saving Mothers, Giving Life; USG, U.S. Government. Source: Adapted from Saving Mothers, Giving Life.^57^

The SMGL theory of change model was built on a district health systems strengthening approach. It was designed to surmount the critical demand- and supply-side delays that prevent women and newborns from receiving lifesaving care in a timely manner, while strengthening the capacity and resilience of the health care system (Figure 1).^15^

The SMGL theory of change was built on a district health systems strengthening approach.

The governments of Uganda and Zambia, their public health systems, the PEPFAR- and USAID-supported maternal and child health platforms, and private for-profit and nonprofit providers were critical inputs and served as the foundation for SMGL's contributions to the district maternity care system. Evidence-based interventions were designed to address all key delays, be context-specific, and strengthen the capacity of the district health system. Four outcomes were anticipated: (1) increased use of services and improved self-care, (2) timelier access to appropriate care, (3) improved quality and experience of care, and (4) a more robust and resilient district health system. It was hypothesized that if these 4 outcomes were achieved together, SMGL-designated populations would see a substantial decrease in maternal and perinatal mortality.

Implementation of the SMGL theory of change followed 7 organizing principles:

1. Reap system-level synergies by addressing all 3 delays to obtaining lifesaving maternal and newborn care concurrently: delays in seeking appropriate care, delays in reaching services in a timely manner, and delays in receiving quality care at a health facility with the capacity to perform 9 signal emergency obstetric and newborn care (EmONC) functions.^16^^–^^22^
2. Recognize the district health system, which extends from community health workers to district hospitals (and to higher levels of care through referrals), as the primary unit for strengthening capacity.^23^^–^^25^ Potential interventions should be assessed in terms of their contributions to improving the functioning of the entire district-level system.
3. Apply a “whole market approach,” which requires identifying and including both public and private inputs (e.g., providers, delivery systems, stakeholders) in planning, execution, and evaluation in a designated district. Together they form the district maternity safety net.
4. Focus on improving services during the most vulnerable period for mothers and newborns—labor, delivery, and early postpartum. Interventions at this time have the possibility of saving the lives of mothers and newborns and preventing fresh stillbirths. The level of fresh stillbirths is often seen as an indicator of the quality of care during labor and delivery.
5. Strengthen the capacity of the health care system to provide comprehensive emergency obstetric and newborn care (CEmONC) within 2 hours of travel time from home or a delivery site for all pregnant women, approximately 15% of whom will experience a life-threatening complication, many without clear predictors.^26^^,^^27^
6. Integrate maternal and newborn health (MNH) services with other reproductive health services, including (1) HIV counseling and testing services to maximize identification and treatment of seropositive pregnant women and prevent mother-to-child transmission, and (2) postpartum family planning for women wishing to delay their next pregnancy.
7. Count, analyze, and report all maternal and perinatal deaths along with the cause of death; improve completion of facility records and registries; institutionalize maternal and perinatal death surveillance and response (MPDSR) in each district and foster high-level awareness of these reviews among traditional, religious, and political leadership to learn from each preventable death and promote necessary health system and cultural changes.

## COUNTRY CONTEXT

In 2011, Uganda and Zambia were chosen as the first SMGL-supported countries based on (1) their interest to the Global Health Initiative; (2) high levels of maternal mortality—MMR of 420 in Uganda and 262 in Zambia in 2010^1^; (3) solid MOH commitment to decreasing maternal and newborn mortality, as evidenced by their Roadmap to Accelerate Reduction of Maternal and Neonatal Mortality and Morbidity and Campaign to Accelerate the Reduction of Maternal, Newborn, and Child Mortality in Africa plans; and (4) the existence of robust PEPFAR- and USAID-supported maternal and child health platforms.^28^^–^^30^ Direct causes of maternal deaths were similar in both countries, with postpartum hemorrhage being the leading cause followed by preeclampsia/eclampsia, sepsis, obstructed labor/ruptured uterus, and complications of unsafe abortions.^1^ The most deadly indirect causes were malaria and HIV.^29^^,^^31^

Inadequate skilled human resources for health were a major constraint to providing effective coverage in both countries.^29^^,^^31^ When SMGL began, the human resources vacancy rate at health facilities in SMGL-supported districts was 40% in both Uganda and Zambia.^11^^,^^12^^,^^32^^–^^34^ Uganda and Zambia also shared high HIV rates (7% and 12% among adults ages 15 to 49, respectively) and their total fertility rates were among the highest in the world (6.2 for both countries) (Table 2). Less than half of births in Zambia, and 57% in Uganda, were attended by skilled birth attendants and the cesarean delivery rates were low at 5% in Uganda and 3% in Zambia. Neonatal mortality rates were 27 and 34 per 1,000 live births in Uganda and Zambia, respectively (Table 2).

**TABLE 2.** Uganda and Zambia National-Level Indicators at the Start of the SMGL Initiative

Indicator	Uganda	Zambia
Maternal mortality ratio (per 100,000 live births)	420^a^	262^a^
Deliveries in facilities	57%^b^	48%^c^
Births by cesarean delivery	5%^b^	3%^c^
Birth attended by skilled birth attendant	57%^b^	47%^c^
Antenatal care coverage: at least 4 visits	48%^b^	60%^c^
HIV prevalence among adults 15–49	7%^d^	12%^d^
Pregnant women with HIV receiving antiretroviral therapy	61%^d^	93%^d^
Total fertility rate	6.2^b^	6.2^c^
Modern contraceptive prevalence rate among all women 15–49	21%^b^	25%^c^
Neonatal mortality rate (per 1,000 live births)	27^b^	34^c^

Abbreviation: SMGL, Saving Mothers, Giving Life.

a 2010 data from *Trends in Maternal Mortality: 1990 to 2015. Estimates by WHO, UNICEF, UNFPA, World Bank Group and the United Nations Population Division* (https://www.who.int/reproductivehealth/publications/monitoring/maternal-mortality-2015/en/).

b 2011 data from *Uganda Demographic and Health Survey 2011* (https://dhsprogram.com/pubs/pdf/FR264/FR264.pdf).

c 2007 data from *Zambia Demographic and Health Survey 2007* (https://www.dhsprogram.com/pubs/pdf/FR211/FR211[revised-05-12-2009].pdf).

d 2011 data from UNAIDS AIDSinfo (http://aidsinfo.unaids.org/).

## PROJECT DESIGN, IMPLEMENTATION, AND ASSESSMENT

### SMGL Learning Districts

**FIGURE 2:**
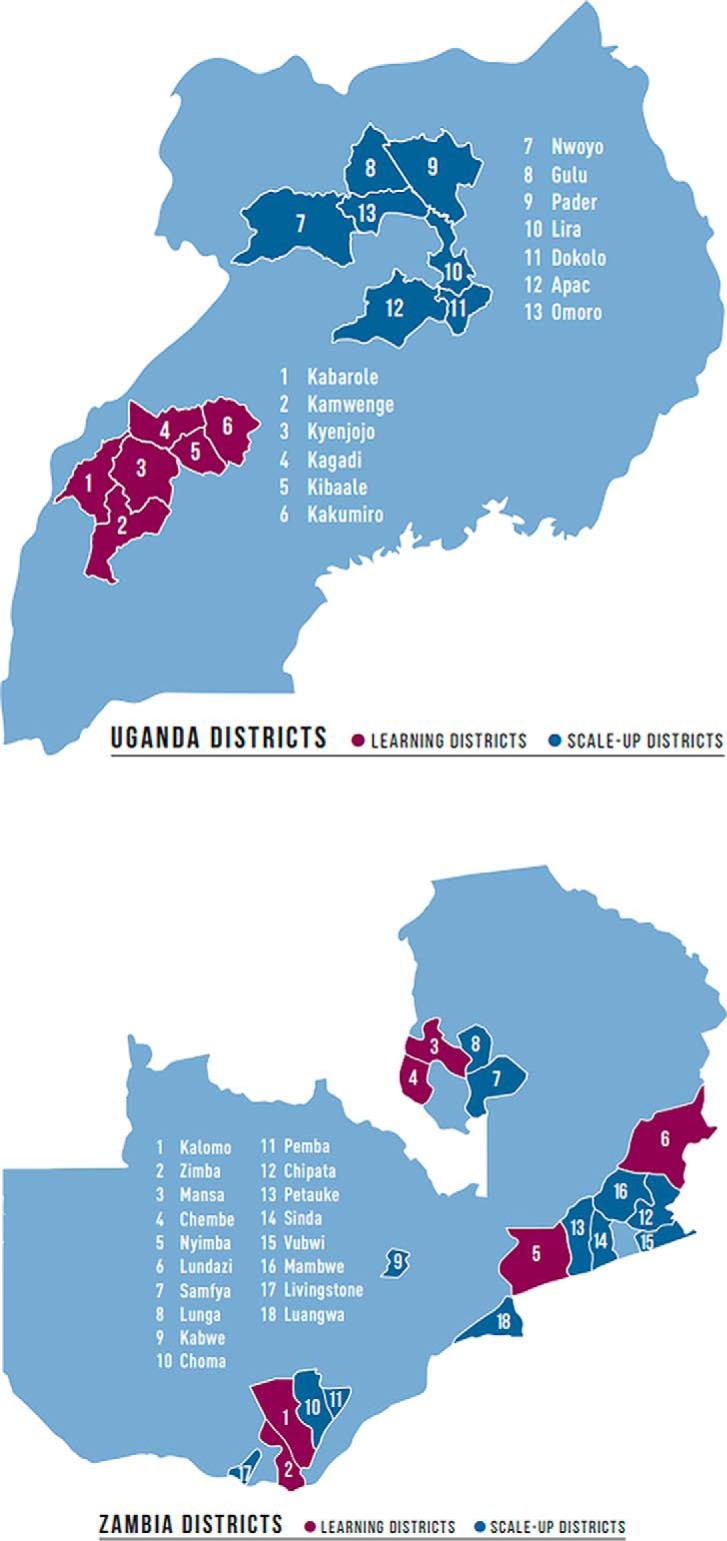
Saving Mothers, Giving Life-Designated Learning and Scale-Up Districts in Uganda and Zambia Source: Adapted from Saving Mothers, Giving Life.^57^

Four districts each in Uganda and Zambia were selected for SMGL support by their MOH based on the large numbers of deliveries and maternal deaths, the availability of existing implementing partners working in the district, and national priorities. The 8 districts in total, designated as the SMGL learning districts, were mostly rural and poor.^8^^,^^11^^,^^12^^,^^30^^,^^31^ Figure 2 shows the learning districts and the scale-up districts. Over the life of the initiative, the 4 learning districts in each country were administratively split further to total 6 learning districts in each country.

In Zambia, the 4 initial learning districts were spread across the country with 2 in Eastern Province (Nyimba and Lundazi), 1 in Southern Province (Kalomo), and 1 in Luapula Province (Mansa). The 4-district population was 880,000 with 46,157 deliveries in 2011. Throughout the initiative, 110 health facilities were engaged, 94% public and 6% private, including 16 health posts, 88 health centers, and 6 hospitals.^11^^,^^35^ Uganda's SMGL-supported districts (Kyenjojo, Kamwenge, Kabarole, and Kibaale, aka “the 4Ks”) were contiguous and located in Western Uganda. The population in the 4Ks was 1.75 million with 78,400 deliveries in 2011. Throughout the initiative, 105 delivering facilities, 61% public and 39% private (18 health centers II, 70 health centers III, 11 health centers IV, and 6 hospitals), were supported by SMGL.^12^^,^^36^

### SMGL Phases

The SMGL initiative was divided into 3 phases: Phase 0—design and startup (June 2011 to May 2012), Phase 1—proof of concept (June 2012 to December 2013), and Phase 2—scale-up and scale-out (January 2014 to October 2017).

#### Phase 0: Design and Startup

**Initiative design.** Design of the SMGL district health systems strengthening approach began in mid-2011 under the aegis of the Global Health Initiative. The Global Health Initiative convened a design team of MNH and HIV technical experts in project development, implementation, costing, policy formulation, and monitoring and evaluation. The aim was to create a highly visible, bold initiative that would galvanize global action and financial support. A draft SMGL model was developed, guided by GHI principles and informed by extensive examination of the evidence base and modeling from the Lives Saved Tool (LiST). (Supplement 1) A goal was established to reduce maternal mortality in SMGL-supported facilities in Uganda and Zambia by 50% in 1 year and an implementation plan was formulated. A notable feature of the plan was that partner funding for SMGL implementation was only guaranteed for an initial 12-month period; if performance was deemed subpar, funding for SMGL could end.

After country and district selection, the U.S. ambassadors for Uganda and Zambia assigned coordination roles to U.S. agency heads (USAID mission director, CDC director, PEPFAR coordinator, Peace Corps lead, and Department of Defense liaison), and interagency working groups were formed. The working groups collaborated with national, provincial, and district MOH-designated SMGL leads (usually district health officers) and implementing partners, forming SMGL country teams. The country teams initially met weekly and then monthly to develop plans and leverage existing partner programs and capabilities. Country teams then created intensive 1-year workplans for the pilot districts in Uganda and Zambia based on addressing the 3 delays and strengthening the system.

The rapid design and execution of the initial SMGL 1-year plan required the participation of existing implementing partners working in SMGL-selected districts. Between Uganda and Zambia, 39 implementing partners were identified, most with set workplans and deliverables (Supplement 2). Under the leadership and supervision of MOH district health management teams and district health and medical officers, extant implementing partner workplans were adapted to support SMGL country and district plans.

**Evaluation design.** The ability to assess and report health outcomes resulting from SMGL efforts required robust evaluation. The headquarters monitoring and evaluation (M&E) committee, composed of specialists from CDC and USAID, developed an ambitious evaluation plan for Phase 1 that was endorsed by the ministries of health and implementing partner representatives in both countries.^37^ The plan included ongoing enumeration of all maternal deaths with verbal autopsies to ascertain cause of death. (See the article by Serbanescu and colleagues from the SMGL supplement.^38^)

Thirty-one indicators were selected for monitoring care at all delivering facilities through quarterly record and registry reviews in SMGL-supported districts in Uganda and Zambia (Supplement 3). In Uganda, these data were collected through Pregnancy Outcomes Monitoring Studies; data were also gathered and displayed monthly at selected SMGL facilities in Uganda using a simple matrix referred to as “BABIES” (Birthweight by Age-at-Death Boxes for Intervention and Evaluation System), which provided short-loop feedback to improve newborn care. Formative special studies^37^ included a qualitative study of women's and communities' perceptions of childbirth in Zambia and a 2-hour travel-time mapping study in Uganda.^39^ (See the article by Schmitz and colleagues from the SMGL supplement.^40^)

SMGL developed a robust evaluation plan that included ongoing enumeration of all maternal deaths with verbal autopsies to ascertain cause of death.

**Baseline assessment.** During Phase 0, baseline studies were undertaken in the 8 learning districts. MMRs were measured through a census with verbal autopsies of deaths among women of reproductive age in Zambia and a Reproductive Age Mortality Survey (RAMOS) in Uganda. (RAMOS uses a variety of sources to identify all deaths of women of reproductive age and decide which of these are maternal- or pregnancy-related.) Health facility assessments (HFAs) of capacity and readiness of the system to provide 9 lifesaving signal functions were undertaken in all public and private delivering facilities in the SMGL-supported districts (Table 3). This enabled planners and implementers to take stock of the existing availability of basic and comprehensive emergency obstetric and newborn care. HFAs were carried out at 3 time points during SMGL: (1) at baseline, to inform SMGL planning and design and to identify needed investments; (2) at the end of the pilot year in 2013 to gauge progress and inform funding and operational decisions during subsequent years; and (3) at endline in 2017 to assess outcomes.

**TABLE 3.** Emergency Obstetric and Newborn Care 9 Signal Functions

Basic Services	Comprehensive Services
1. Administer parenteral antibiotics	Perform signal functions 1 through 7 plus:
2. Administer uterotonic drugs (i.e., parenteral oxytocin, misoprostol)	8. Surgery (cesarean delivery)
3. Administer parenteral anticonvulsants for preeclampsia (i.e., magnesium sulfate)	9. Blood transfusion
4. Manually remove the placenta	
5. Remove retained products of conception (e.g., manual vacuum extraction, misoprostol, dilation and curettage)	
6. Perform assisted vaginal delivery (e.g., vacuum extraction, forceps delivery)	
7. Perform basic neonatal resuscitation (e.g., bag and mask)	

Source: WHO, UNFPA, UNICEF, and Mailman School of Public Health.^27^

Common gaps identified from the baseline HFA included the following:

- **Delay 1: Demand.** The number of Government-established community health workers, village health teams (VHTs) in Uganda and Safe Motherhood Action Groups (SMAGs) in Zambia, was inadequate. Women booked late for antenatal care visits and attendance of 4 or more antenatal care visits was low (46% in Uganda).^41^
- **Delay 2: Access.** Women had limited access to comprehensive CEmONC facilities within 2 hours (only 51% to 55% of women were able to reach CEmONC within 2 hours using motorized vehicles) due to few operating theaters and blood banks, and lack of transport vehicles and referral protocols. Maternity waiting homes were often dilapidated and deserted.
- **Delay 3: Quality.** Many maternity blocks in hospitals and health centers were run-down and overcrowded, and they lacked water, electricity, and functioning toilets. Equipment was missing, inoperative, or insufficient for the client load. Facilities lacked 24-hour staffing of skilled birth attendants, anesthetists, and surgeons.
- **Health Systems Strengthening.** In the face of limited quality improvement activities, facilities experienced frequent drug and supply stock-outs and weak capture, analysis, and reporting of health outcome data.

These gaps and other district-specific challenges were addressed in SMGL district workplans.

**Startup.** Startup activities began early in 2012. At the national level in Uganda and Zambia, routine meetings were held with the interagency working groups, MOH representatives, and implementing partners. Preparations for work with private providers through the Programme for Accessible Health Communication and Education (PACE) project and Marie Stopes International were initiated in Uganda. In Zambia, where the SMGL learning districts were spread out across the country, SMGL district coordinators—often retired midwives—were hired to harmonize all SMGL activities in their district with district health officers and district health management teams, and to serve as a link with implementing partners. During this phase, training commenced for providers and existing government-sponsored community health workers—SMAGs and VHTs. These health workers were recruited from the local community. Groups were a mix of men and women and often included former traditional birth attendants. SMGL provided these volunteers with resources such as gumboots, flashlights, T-shirts, and bicycles. In Zambia, Peace Corps volunteers were recruited and trained as community mobilizers to work with SMAGs to increase demand and organize community transport systems. By the end of the initiative, SMGL-dedicated Peace Corps volunteers were in all 18 SMGL-supported districts.

To address barriers to timely access to care, SMGL brought lifesaving care closer to women, decreased travel time to appropriate care, and brought women closer to emergency services.

#### Phase 1: Proof of Concept

Results for Phase 1 are based on data for the 12-month period from June 2012 through May 2013. Analysis and write-up of lessons, however, continued through December 2013.

**Interventions.** District-level MOH staff led the implementation process working with implementing partners funded by PEPFAR, CDC, USAID, and Merck. In the learning districts, the following interventions were carried out to address the health system gaps identified in the Phase 0 HFAs, by delay, in accord with the SMGL theory of change.

- **Delay 1: Demand.** Tackling this delay required not only effecting change in individual behaviors but also influencing community norms. SMAGs and VHTs identified pregnant women and initiated antenatal home visits covering all villages across the 8 learning districts. They provided childbirth education and anticipatory guidance. Specific topics included: self-care and a healthy diet, attending antenatal and postnatal care visits and delivering in a facility, family planning, recognition of maternal and newborn danger signs, being tested for HIV, and undertaking birth planning and saving to cover the costs of transport and medical care. Messages given during these family visits were reinforced by including husbands and household members, holding community sensitization meetings, and training traditional leaders to be “change champions.” Multimedia campaigns, which included community sensitization skits, radio announcements, community documentary screenings, and billboards, were also fielded in both countries. In Zambia, Peace Corps volunteers trained SMAGs on a home-visit protocol using the national SMAG curriculum. (See the article by Serbanescu and colleagues from the SMGL supplement.^41^)
- **Delay 2: Access.** A travel-time study in Uganda and HFA results from both countries confirmed that timely access to care was a major problem in all 8 SMGL-supported districts. SMGL programming addressed this problem in 3 ways: bringing lifesaving care closer to women, decreasing travel time to appropriate care, and bringing women closer to emergency services. Select maternity wards and surgical theaters were refurbished to upgrade facility capacity and optimize 2-hour access to CEmONC care. In Uganda, subsidized motorcycle transport vouchers and private-care vouchers, distributed by VHTs, were rapidly scaled up during Phase 1. In Zambia, where long distances to care are the norm, maternity waiting homes were refurbished or built next to EmONC facilities by the Department of Defense and the Merck-led Maternity Waiting Home Alliance. In both countries, SMGL ensured appropriate communication tools, such as cell phones and radios, and district-specific protocols to facilitate transfers. (See the article by Ngoma et al. from the SMGL supplement.^42^)
- **Delay 3: Quality.** Baseline HFA results had revealed the need for significant improvements in the quality of services provided if women were to receive lifesaving care for complications; many aspects of the health system would need to be strengthened. Efforts to improve the quality of services engaged frontline health care providers and facility managers. SMGL hired strategically placed midwives, nurses, anesthetists, and doctors (147 providers in Uganda and 19 in Zambia). In both countries, many of the midwives hired were retired, seasoned health professionals. In Uganda, staff was hired with the understanding that their positions would be picked up by the MOH when SMGL funding ended. SMGL doctors received increases in their salaries to work in rural health center IVs rather than hospitals, an incentive that was subsequently adopted nationally by the MOH. Quality improvement committees were formed and the BABIES matrix was introduced into all EmONC facilities. Quality improvement committees were trained to sensitize providers on the importance of respectful care. Merck for Mothers worked through the PACE project to provide technical assistance to private providers in order to upgrade their skills. Health care providers in both countries were trained by MOH trainers and routinely mentored on EmONC, Helping Babies Breathe, essential newborn care, uterine balloon tamponade (Zambia), maternal and perinatal death reviews, syphilis screening, prevention of mother-to-child transmission of HIV, infection prevention, and operative skills. Obstetricians and gynecologists associations in both countries provided clinical mentoring to district medical officers and district health officers in SMGL-designated districts and the professional societies were in turn strengthened with technical assistance from the American College of Obstetrics and Gynecology. Project C.U.R.E. supplied donated facility-specific, essential equipment and commodities (including hospital and delivery beds, surgical tables and lights, resuscitation supplies, sterilization equipment, sutures, and gloves), shipping 16 containers to Uganda and 20 to Zambia over the life of the initiative. (See the article by Morof et al. from the SMGL supplement.^43^)
- **Health systems strengthening.** Activities to strengthen the health system included providing HIV-related diagnostics and treatment and family planning services at the same location and times as MNH services to create “one-stop” shops. Both countries followed the Option B+ HIV treatment guidance, which supports HIV testing and counseling during antenatal care and offering women found to have HIV infection lifelong antiretroviral therapy. This facilitated the SMGL HIV testing and treatment approach: pregnant women were tested for HIV during antenatal care visits, and if seropositive, midwives were empowered to place them on antiretroviral therapy to protect the life of the mother and prevent mother-to-child transmission. In select SMGL-supported districts, providers were trained to provide postpartum family planning. District medical and health officers and in-charges received instruction on drug logistics and forecasting to prevent chronic stock-outs of essential medicines. Facilities were equipped with rainwater catchment systems, solar panels, and functioning toilets. Maternal and perinatal deaths were reviewed through routine maternal and perinatal death surveillance and response efforts in facilities. The CDC provided capacity strengthening of district-level teams on monitoring and evaluation. SMGL staff supported monthly district-led data reviews of MNH indicators, quarterly provincial-level reviews, and strengthening of the District Health Information Management System (DHIS2), a free and open-source health management data platform. (See the article by Serbanescu and colleagues from the SMGL supplement.^38^)
- **Data collection activities.** After 12 months of Phase 1 implementation (June 2012 to May 2013), endline Phase 1 studies were conducted in the 8 learning districts to assess the status of the SMGL indicators and thus gauge progress at the end of year 1.^17^ In addition, a mixed-methods external implementation evaluation of Phase 1 was undertaken by Columbia University.^29^ This evaluation examined the reach, extent, fidelity, and dynamic effects of the initiative in order to identify best practices and remaining barriers to reducing maternal mortality. Data from these evaluations were analyzed and results were reported at an SMGL global dissemination meeting in January 2014.^44^^,^^45^ (See Supplement 4.)

Health systems strengthening activities included providing HIV-related diagnostics and treatment and integrated family planning services.

#### Phase 2: Scale-Up and Scale-Out

Early in 2014, the partners met to examine SMGL performance and to modify SMGL's approach, governance, assessment, and implementation for Phase 2. These adjustments are described in the following sections.

**Initiative.** The partners decided to maximize the return on initial investments in Uganda and Zambia by committing to operate in both countries until October 2017. SMGL would aim to achieve near-national coverage of the SMGL approach in Uganda and Zambia, defined by the partners as ≥70% population coverage, and would select 1 additional country for SMGL implementation. In 2015, Nigeria became the third and final SMGL country.^46^ There, the SMGL systems approach was rolled out across Cross River State (population 3.7 million) and will be supported until October 2019. The governing partners for SMGL Nigeria are USAID Washington, USAID Nigeria, Merck for Mothers, and Project C.U.R.E.

**Governance.** MOH representatives were invited to join the Leadership Council, SMGL's global governing body, and partners agreed to re-examine their resource pledges and submit quarterly contribution reports.

**Scale-up district assessment.** The SMGL partners agreed that due to the high cost and management burden of undertaking detailed information gathering, a limited number of M&E activities would take place in the scale-up districts of both countries. The focus of these efforts would be to guide program adjustments for quality improvement: HFAs at baseline to inform initial programming, quarterly record and registry data gathering at CEmONC facilities only, and Health Management Information System reporting on indicators of interest for all facilities on a quarterly basis. (See the article by Isabirye et al. from the SMGL supplement.^47^)

**Implementation.** Interventions introduced in Phase 1 were largely maintained with a few exceptions: Mama Pack distribution in Zambia ended based on concerns about sustainability; repair or replacement of 2-way radios became unnecessary as the availability of cell phones increased; and ongoing enumeration of maternal deaths by Zambia SMAGs was discontinued after problems with data gathering during the proof-of-concept phase. VHTs in Uganda continued ongoing enumeration. The partners endorsed several context-specific programmatic changes for the learning districts and the scale-up districts. The SMGL time frame of interest was lengthened from intrapartum through 24 hours postpartum to 48 hours postpartum in Uganda and 72 hours postpartum in Zambia to conform to host country guidelines and enable greater focus on postpartum family planning and postnatal care. In the face of nonsignificant reductions in pre-discharge neonatal mortality in Uganda during Phase 1, SMGL increased programming for newborns. Additional interventions included: ensuring availability of newborn corners (flat surfaces for newborn resuscitation) in each delivery room; opening neonatal special care units, and Kangaroo Mother Care units in 8 health center IVs and 3 hospitals where stable low birth weight and premature newborns could be cared for; upgrading the existing neonatal intensive care unit at Fort Portal Regional Referral Hospital; increasing training and drilling on newborn resuscitation and essential newborn care; and implementing the BABIES matrix in additional facilities. (See the article by Morof and colleagues from the SMGL supplement.^43^)

In Zambia, where postpartum hemorrhage was the leading cause of maternal death and in the context of long distances to delivery care, 3 interventions were prioritized for Phase 2: (1) constructing and refurbishing maternity waiting homes, (2) introducing and institutionalizing uterine balloon tamponade, and (3) strengthening the national blood transfusion system. Maternity homes, located next to SMGL-supported EmONC facilities, were built or refurbished by the U.S. Department of Defense or under the Maternity Waiting Home Alliance. (See the article by Ngoma and colleagues from the SMGL supplement.^42^) SMGL helped drive policy changes that allowed uterine balloons to be placed by nurses and midwives and for uterine balloon tamponade to be included in the national EmONC curriculum. Across the 18 Zambian SMGL districts, providers were trained in the assembly and use of uterine balloon tamponade. With funds from SMGL and the government of Zambia, several district blood bank hubs were established to provide 24-hour blood testing and availability of fresh-frozen blood and plasma.

SMGL increased programming for newborns during the second phase, including opening neonatal special care units and increasing training on newborn resuscitation.

**Contextual changes.** Important contextual changes occurred during Phase 2 at the district level. The 4 original learning districts in Uganda were divided into 6 districts—Kabarole, Kibaale, Kamwenge, Kyenjojo, Kakumiro, and Kagadi—and 7 new scale-up districts were added in the north—Nwoya, Gulu, Omolo, Pader, Lira, Apac, and Dokolo. All were selected by the MOH. Due to a change in the implementing partner for the new SMGL Uganda northern districts, full execution of the SMGL approach did not begin until 2015 and ended 2 years later. (Project description and results can be found in the article by Isabirye and colleagues from the SMGL supplement.^47^) In Zambia, the 4 learning districts were divided into 6 districts through an administrative re-districting process—Nyimba, Lundazi, Kalomo, Zimba, Mansa, and Chembe—and 12 additional districts were added across the country—Samfya, Lunga, Kabwe, Choma, Pemba, Chipata, Petauke, Sinda, Vubwi, Mumbwa, Livingstone, and Luangwa (Figure 2).

**Endline evaluation studies.** After the Phase 1 endline studies showed a 35% reduction in facility maternal mortality and positive results for process and quality indicators in the SMGL-supported learning districts in both countries, a summative evaluation plan was developed by the M&E committee and the SMGL Secretariat. The plan was endorsed by the SMGL Leadership Council members who also pledged funding for executing the plan. Using 2016 as the index year for SMGL final results, end-of-initiative studies were undertaken in 2017 to establish outcomes in the learning districts: (1) a census in Zambia and a RAMOS in Uganda,^38^^,^^48^ (2) repeat HFAs in all delivering facilities in the learning districts,^38^ (3) a cost-effectiveness study addressing the 3 delays,^7^ (4) a secondary analysis comparing SMGL district outcomes with findings from the Uganda Demographic and Health Survey (DHS) in comparison districts and nationally,^31^ (5) a follow-on qualitative study of community perspectives on childbearing in Zambia,^49^ and (6) a repeat travel-time mapping study in Uganda to gauge if the SMGL initiative resulted in greater access to care.^50^

## RESULTS

### Key Health Facility and Population-Based Assessment Results

Select results from Phase 1 have previously been reported.^7^^,^^17^^,^^29^^,^^39^^,^^51^^–^^53^ What follows is an overview of key results at baseline and 2016 endline for the SMGL-supported learning districts. Table 4 compares selected baseline and endline indicators by type. A description of data collection methods, indicators, and baseline and endline results are included in the article by Serbanescu and colleagues from the SMGL supplement.^38^ A comparison of SMGL outcomes with those from DHS and UN maternal mortality estimates is presented in Supplement 5.

**TABLE 4.** Key Results at Baseline and Phase 2 Endline in the SMGL Learning Districts

SMGL Indicator	Uganda				Zambia			
	2012 Baseline	2016 Phase 2 Endline	% Change Baseline to Phase 2	Significance^a^	2012 Baseline	2016 Phase 2 Endline	% Change Baseline to Phase 2	Significance^a^
**GOAL**									
Institutional MMR (per 100,000 live births)	534	300	−44	***		370	231	−37.6	***
Community MMR (per 100,000 live births)	452	255	−44	***		480	284	−40.8	***
Pre-discharge neonatal mortality rate (per 1,000 live births)	8.4	7.6	−10	NS		7.7	8.7	+14	NS
Institutional perinatal mortality rate (per 1,000 births)	39.3	34.4	−13	***		37.9	28.2	−26	***
Institutional total stillbirth rate (per 1,000 births)	31.2	27.0	−13	***		30.5	19.6	−36	***
**DEMAND**									
Health facilities that report having a VHT (Uganda) or SMAG (Zambia) (%)	18	92	+400	***		64	93	+46	***
Institutional delivery rate (%)	46	67	+47	***		63	90	+44	***
Deliveries in EmONC facilities (%)	28	41	+45	***		26	29	+12	***
Deliveries in lower-level facilities (health center II, III) (%)	17	26	+48	***		37	61	+67	***
**ACCESS**									
Facilities that report having an associated mother's shelter (%)	0	4	NA	NA		29	49	+69	***
Institutional deliveries supported by transport vouchers (%)	6	24	+277	***		Vouchers not provided in Zambia			
Number of BEmONC facilities where the 7 signal functions were performed in last 3 months	3	9	+200	NA		3	8	+167	NA
Number of CEmONC facilities where the 9 signal functions were performed in last 3 months	7	17	+143	NA		4	5	+25	NA
24/7 services at health centers (%)	75	89	+18	NS		65	96	+41	***
**QUALITY OF CARE**									
Facilities reporting having performed newborn resuscitation in the previous 3 months (%)	34	88	+155	***		27	75	+173	***
Facilities providing active management of the third stage of labor (%)	75	96	+28	***		72	96	+33	***
Population-based cesarean delivery rate (%)	5.3	9.0	+71	***		2.7	4.8	+79	***
Hospitals that currently have at least 1 long-acting family planning method (%)	63	94	+51	**		50	75	+50	NS
Number of women receiving PMTCT treatment	1262	2155	+71	NA		930	1036	+11	NA
**HEALTH SYSTEMS STRENGTHENING**									
Hospitals conducting maternal death audits or reviews (%)	31	94	+201	***		50	100	+100	NA
Health facilities that did not experience stock-outs of oxytocin in the last 12 months (%)	56	82	+46	***		75	75	−0.4	NS
Health facilities that did not experience stock-outs of magnesium sulfate in the last 12 months (%)	48	64	+34	***		20	43	+115	***

Abbreviations: EmONC, emergency obstetric and newborn care; BEmONC, basic emergency obstetric and newborn care; CEmONC, comprehensive emergency obstetric and newborn care; MMR, maternal mortality ratio; NA, not applicable; NS, nonsignificant; SMAG, Safe Motherhood Action Group; VHT, Village Health Team; PMTCT, prevention of mother-to-child transmission of HIV.

a *** *P* <.01; ** *P* <.05; * *P* <.10. NA in cases where significance testing was not warranted.

Source: Serbanescu et al.^38^

#### Demand

The chances of surviving childbirth are improved when a woman gives birth in a facility, attended by a skilled birth attendant.^54^^–^^56^ Over the life of SMGL, the institutional delivery rate, or the proportion of births occurring in delivery facilities, increased from 46% to 67% in Uganda (a 47% increase) and from 63% to 90% (a 44% increase) in Zambia SMGL-supported facilities.

The institutional delivery rate increased by 47% in SMGL-supported facilities in Uganda and by 44% in Zambia.

#### Timely Access

SMGL prioritized bolstering the system's capacity to provide timely lifesaving emergency care. The number of facilities that performed all 7 signal functions that constitute basic emergency obstetric and newborn care (BEmONC) increased from 3 to 9 in Uganda (200%) and from 3 to 8 in Zambia (167%). Similarly, the number of CEmONC facilities increased from 7 to 17 (143%) in Uganda and from 4 to 5 (25%) in Zambia.

In 3 SMGL-supported districts in Uganda, transportation vouchers enhanced women's access to essential and emergency health services by covering the cost of motorcycle rides to facilities for delivery, 4 antenatal care visits, and 1 postnatal care visit. In 2016, almost 1 out of 4 women who delivered in SMGL facilities used transportation vouchers to reach care. In Zambia where motorcycle transport is not generally available, maternity waiting homes were built or upgraded to provide mothers a safe place to stay near an EmONC facility during the last weeks of pregnancy. The proportion of SMGL facilities that reported having an associated maternity waiting home increased significantly from 29% at baseline to 49% at endline (a 69% increase).

Cesarean delivery rates increased by 71% in SMGL-supported districts in Uganda and by 79% in Zambia.

#### Quality

The range of interventions that SMGL implemented to enhance quality of care largely proved effective:

- Population-based cesarean delivery rates increased by 71% (from 5.3% to 9.0%) in Uganda and 79% (from 2.7% to 4.8%) in Zambia in SMGL-supported districts. The rates achieved are still below the World Health Organization (WHO) recommended rates of 10% to 15%. (Regardless of the rate, cesarean deliveries should be performed only when medically indicated).
- The percentage of facilities reporting having performed newborn resuscitation in the last 3 months increased by 155% (from 34% to 88%) in Uganda and by 173% (from 27% to 75%) in Zambia.
- The percentage of all SMGL-supported facilities in Uganda that reported active management of the third stage of labor increased by 28% (from 75% to 96%). In Zambia, the change from baseline was 33% (72% to 96%).
- Having at least 1 long-acting reversible family planning method in SMGL-supported facilities increased in both counties. In Uganda, availability increased by 51% (from 63% to 94%) of facilities. In Zambia, it improved by 50% (from 50% to 75%) of facilities.
- The percentage of hospitals conducting maternal death audits tripled in Uganda (from 31% to 94%) and doubled in Zambia (from 50% to 100%).
- The number of HIV-seropositive women who received prophylaxis or treatment for the prevention of mother-to-child transmission increased by 71% in Uganda, from 1,262 to 2,155 women, and by 11% in Zambia, from 930 to 1,036 women (denominators not available).

#### Health Systems Strengthening

Access to medications was positive but uneven. While SMGL funds were not used to procure medicines in Phase 2, providers were trained in supply chain management. The proportion of all health facilities that did *not* experience stock-outs of oxytocin in the last 12 months increased by 46% (from 56% to 82%) in Uganda but did not change in Zambia (from 75% to 75%). The proportion of all health facilities that did *not* experience stock-outs of magnesium sulfate in the last 12 months increased significantly in both countries, by 34% (from 48% to 64%) in Uganda and by 115% in Zambia (from 20% to 43%).

#### Impact

From baseline to endline (2012–2016), the MMR declined by 44% in both facilities and districtwide in Uganda (from 534 to 300 per 100,000 live births in facilities and from 452 to 255 in the community). MMR declined by 38% in SMGL-supported facilities in Zambia (from 370 to 231) and by 41% districtwide (from 480 to 284). All declines were statistically significant.

In Uganda, the perinatal mortality rate declined by 13% in SMGL-supported facilities (from 39.3 to 34.4 perinatal deaths per 1,000 births). The total institutional stillbirth rate also declined by 13% (from 31.2 to 27.0 per 1,000 births). Both values are statistically significant. The pre-discharge neonatal mortality rate fell by 10% (from 8.4 to 7.6 per 1,000 live births); however, this was a nonsignificant change. In Zambia, the institutional perinatal mortality rate declined by 26% in SMGL-supported facilities (from 37.9 to 28.2) and the institutional stillbirth rate declined by 36% (from 30.5 to 19.6). Both declines were significant. The change in the pre-discharge neonatal mortality rate was not significant at +14% (from 7.7 to 8.7).

The MMR declined significantly in both SMGL-supported facilities and districts in Uganda and Zambia.

#### Public and Private Health Care Facilities

In Uganda, where 40% of facilities receiving SMGL support were private, the endline evaluation explored in a separate analysis whether any differences existed in the impact indicators by the type of sector providing delivery care (Table 5). The majority of SMGL facility deliveries occurred in public facilities (83.4% public vs. 16.6% private). The proportion of women who delivered by cesarean delivery was slightly lower in public-sector facilities compared with the private sector (13.0% vs. 15.7%, respectively) (data not shown). Generally, no significant differences existed in the occurrence of adverse pregnancy outcomes among women delivering in the private and public sectors in 2016 in Uganda, with the exception of the intrapartum stillbirth rate, which was higher in private facilities than in public facilities (17.0 vs. 13.8 per 1,000 births, respectively). See Supplement 6 for more information about private-sector activities in Uganda.

**TABLE 5.** Select Indicators by Delivery Care Service Sector in Uganda, 2016

Indicator	Public-Sector Facilities	Private-Sector Facilities	Significance^a^
Maternal mortality ratio (per 100,000 live births)	301	295	NS
Direct case fatality rate	1.8	1.5	NS
Perinatal mortality rate (per 1,000 births)	34.0	36.4	NS
Intrapartum stillbirth rate (per 1,000 births)	13.8	17.0	**
Total stillbirth rate (per 1,000 births)	26.6	28.7	NS
Pre-discharge neonatal mortality rate (per 1,000 live births)	7.6	7.9	NS

Abbreviation: NS, nonsignificant.

a ** *P*<.05.

Source: Serbanescu et al.^38^

## DISCUSSION

The positive results from the SMGL Phase 2 endline evaluation studies (2016 data) in the learning districts in Uganda and Zambia are substantial. However, SMGL's non-randomized, before-and-after design makes it challenging to attribute the outcomes documented after nearly 5 years of implementation solely to the SMGL health systems strengthening approach. The Columbia University implementation evaluation of SMGL's proof-of-concept year did include comparison districts, but there was no randomization. Still, the MMR declined significantly faster in the SMGL-supported learning districts compared with national-level declines. Over a 5-year span the average annual rate of reduction in Uganda learning districts was 11.5% compared with the national rate of 3.5% using DHS values. The difference-in-differences between the drop in MMR in SMGL areas compared with the drop in the MMR nationally is statistically significant (*P* = .02) (Supplement 5).

The findings for Zambia are similar although the timing of the DHS did not allow use of DHS data for comparison. Instead, the UN maternal mortality estimates for Zambia for the period 2011–2015 were used. The average annual rate of reduction in SMGL districts in Zambia was 10.5% vs. a national rate of 2.8%.^1^ These more rapid declines in MMR in SMGL program areas compared with national levels in both countries over a 5-year period suggest that SMGL outcomes are not solely due to secular trends (Supplement 5).

The results of the SMGL evaluation provide answers to some questions that are critical to ending preventable maternal and newborn deaths, while leaving other questions unresolved.

The MMR declined significantly faster in the SMGL-supported districts than nationally.

### Why Does the SMGL Theory of Change Focus on All Pregnant Women Rather Than Only Those Experiencing a Complication?

The 3-delays model, introduced by Thaddeus and Maine in 1994 in their seminal article,^16^ provided a conceptual framework for programming to surmount the key barriers faced by women with obstetric complications. In the SMGL theory of change, we focused on all pregnant women within the SMGL-supported districts because many maternal complications are difficult to predict and prevent, can arise quickly, and can result in a maternal death in a short period of time. The SMGL systems approach aimed to provide access to emergency care within 2 hours from home or a lower-level health facility for all pregnant women in SMGL-supported districts.

### Can a District Health Systems Strengthening Approach Addressing the 3 Delays Contribute to Maternal Mortality Reductions in High-Burden, Low-Resource Countries?

The data show significant reductions in the MMR in the learning districts in both Uganda and Zambia after nearly 5 years of SMGL implementation. The contribution of SMGL to these changes is plausible given the greater rate of reduction in program areas compared with national rates in both countries (Supplement 5). In Uganda, 70% of the total MMR reduction, from baseline to endline Phase 2, occurred during Phase 1, suggesting that once inputs were in place, the systems approach was successful in sustaining the reduced MMR. This is particularly instructive as after Phase 1, in the context of erratic funding flows, implementation was uneven. In spite of these lapses, reductions were sustained over the life of SMGL, based on robust analysis of the SMGL routine quarterly indicators and Pregnancy Outcomes Monitoring Studies values.^57^

### What About Newborn Deaths and Stillbirths?

Decreases in institutional perinatal deaths were statistically significant in Uganda and Zambia at 13% (31.2 to 27.0) and 26% (37.9 to 28.2), respectively. The declines in the total stillbirth rate (fresh and macerated stillbirths) were also significant in both countries (13% in Uganda and 36% in Zambia).^38^ However, changes in pre-discharge neonatal mortality rates were nonsignificant. Further analysis is needed to understand why the SMGL approach was able to decrease stillbirths but not newborn deaths. We hypothesize that, in the past, newborns who were not breathing at birth were laid aside and categorized as stillbirths but that after HBB training some were successfully resuscitated. A portion of these now breathing newborns potentially succumbed to fatal complications. It is also unclear if the public-private differences seen in intrapartum stillbirth rates in Uganda reflect differences in health care provision or in clinical risk factors. (See the article by Serbanescu and colleagues from the SMGL supplement.^38^)

Results from a modeling exercise support the SMGL theory of change that an integrated systems approach addressing both demand- and supply-side barriers is more impactful than individual interventions.

### What Is the Minimum Package of Interventions Needed to Reduce Maternal and Neonatal Mortality?

The SMGL theory of change posits that an integrated systems approach addressing both demand- and supply-side barriers is more impactful than individual and/or uncoordinated interventions, especially for a complex and multifaceted problem such as maternal mortality. The results of the Qualitative Comparative Analysis (QCA) modeling from Uganda support this hypothesis.^58^ The QCA examined the relative power of varied *bundles of interventions* to replicate the Phase 1, first-year achievement of reducing *community* maternal mortality in SMGL-supported districts in Uganda by 30% (*facility* deaths were reduced by 35%) The results suggest that the most powerful bundle of interventions (most effective at lowest cost) was comprised of 4 interventions: VHTs (*demand*); transportation vouchers (*access*); availability of staff (*quality*); and availability of medicines (*health systems strengthening*). If run individually, none of these interventions achieved the 30% MMR reduction, and if the results from these individual interventions were then added together, the sum did not achieve the reduction of the optimal bundle. It appears that it is not only these critical interventions but the synergy created by addressing both supply- and demand-side barriers that accelerates change.^60^ It would be instructive to undertake a QCA study in Zambia to see if similar results are found.

### What About Cost?

SMGL's achievements are often tempered by concerns that the SMGL approach was too expensive for replication. In order to rigorously examine this critical consideration and establish the relative value for money, it is necessary to compare the cost of SMGL implementation with other initiatives that have achieved equivalent health outcomes. Unfortunately, few MNH projects are comparable to SMGL in terms of complexity, robust capture of both facility and districtwide health outcomes (MMR, perinatal mortality rate, neonatal mortality rates, cause of death), and commitment to tallying expenditures.^29^^,^^60^ Even when examining the cost-effectiveness of individual MNH interventions, there is a paucity of high-quality cost-effectiveness studies.^61^^–^^64^ These features have left evaluators without ideal counterfactuals.^65^^–^^67^

To better understand relevant SMGL cost outlays over the life of the initiative, 3 costing studies were undertaken (Supplement 4). All 3 studies projected that after investing in essential capital improvements and streamlining operations, running costs would decrease substantially. Those predictions proved accurate. By design, external funding tranches for SMGL implementation in Uganda and Zambia were decreased yearly while the number of SMGL districts increased, resulting in substantial reductions in funding per learning district over Phase 2. During that same period, maternal health outcomes in the learning districts continued to show improvement.

The endline 3-delays costing study looked at the cost in 2016 of addressing all 3 delays: demand generation, accelerating access to appropriate care including referral, and improving the quality of care at the facility. The expenditure per maternal and perinatal life-year gained was found to be US$177 in Uganda and US$206 in Zambia. These values are inclusive of startup and capital costs—both expressed as annual equivalents. The authors conclude that the SMGL approach is cost-effective, with the cost per life-year gained in Uganda at 25.6% of gross domestic product (GDP) per capita and at 16.4% of GDP per capita in Zambia. Both values are less than 50% of GDP per capita, a benchmark for cost-effectiveness. In terms of affordability, the additional (incremental) costs associated with the SMGL approach would add less than 0.5% to the health spending from GDP in both countries (from 7.3% to 7.5% in Uganda and from 5.4% to 5.8% in Zambia). Recent models suggest that, at a minimum, an additional US$11 per capita per year is necessary to meet the full needs of MNH care in sub-Saharan Africa.^68^ The incremental costs of the SMGL initiative of US$1.36 per person per year in Uganda and US$4.85 per person per year in Zambia are far less than these modeled estimates, and much less than that spent on antiretrovirals per person treated per year, which stood at an average of US$136.80 in 2015.^69^ (See the article by Johns and colleagues from the SMGL supplement.^70^)

### What About Sustainability?

In Uganda and Zambia there is both increased MOH commitment to the health systems strengthening approach and heightened societal awareness that most maternal and newborn deaths can and should be prevented.^29^ Yet, it is likely that ongoing donor funding and technical assistance will be required in the short term to maintain the positive results achieved during SMGL implementation. Below, we look at country capacity and ownership as 2 important domains to gauge the likelihood that key elements of the SMGL health systems strengthening approach will be sustained.

**Country capacity.** Capacity building of district-level medical and public health staff included clinical training, monthly on-site mentoring, and management; data gathering, analysis, reporting, and response; quality improvement; drug logistics; and budget development. Physicians in both Uganda and Zambia were trained (for the first time) on International Classification of Diseases (ICD) 10 Maternal Mortality coding of deaths, a prerequisite skill for a functioning maternal death surveillance and response system and a civil registration and vital statistics system. In both countries, the initiative led to improvements in tracking routine service delivery indicators as part of the national health management and information systems. In 2011, the governments of Uganda and Zambia began using DHIS2 as an electronic platform for aggregated health service data. In both countries, the SMGL-supported districts piloted DHIS2 implementation to collect, store, and analyze data on maternal and reproductive health. The improvements were scaled up to the national level by the end of 2012. Another important activity in Zambia was training SMGL district doctors and nurses in blood transfusion safety. Hospital Transfusion Committees were established to improve monitoring of blood supplies through the use of short message services (SMS or texts) for forecasting and planning to avert shortages. When donor funding recently decreased for blood-safety programs, the government of Zambia increased its health budget to ensure an adequate supply of blood for its citizens. (See the article by Healey et al. from the SMGL supplement.^71^)

Beyond training, SMGL country technical leads were supported to assume leadership positions within SMGL and to provide technical assistance to other SMGL countries. A team of Uganda SMGL leads traveled to Nigeria to provide technical assistance to the Nigeria SMGL team to carry out HFAs in Cross River State health care facilities, public and private, and also to Zambia to support HFAs in Phase 2 scale-up districts. A Zambia SMGL lead traveled to Afghanistan and assisted the USAID Mission to incorporate lessons learned from the SMGL approach into their MNH strategic plan. SMGL country staff prepared posters and presented at the yearly SMGL team-building meetings, and staff members were encouraged to submit abstracts and present at global MNH meetings.

In both Uganda and Zambia, SMGL led to improvements in tracking routine service delivery indicators as part of the national health management and information systems.

**Country ownership.** District health leaders in Uganda reported high levels of ownership of SMGL and cited the addition of key inputs as strategic: filling human resource gaps; strengthening referral systems; expanding the number of CEmONC facilities; improving the supply of blood for transfusion; mentoring health personnel; and increasing demand and access through VHTs, transportation vouchers, and community champions. SMGL also influenced national planning and budgeting for maternal health: the Wage Bill included allowances to support doctors working at health center IVs located in rural areas based on SMGL's remuneration approach; nearly 75% of the midwives hired by SMGL were picked up by the MOH; additional midwifery training was provided for enrolled nurses; and the voucher program laid the groundwork for a national program.^29^ Lessons learned from the SMGL approach were incorporated into the Global Financing Facility Investment Case,^72^ the WHO Quality, Equity, Dignity initiative country plan, and USAID requests for assistance and contracts. Between these initiatives, over half of the Ugandan population will be covered by a district health systems strengthening approach by 2020. (See the articles by Healey et al.^71^ and Palaia et al.^73^ from the SMGL supplement.)

In Zambia, preexisting CDC cooperative agreements with provinces and district-support from CDC and USAID implementing partners enabled early leveraging of funds and increased district ownership of SMGL. SMGL worked with other donors, the Swedish International Development Cooperation Agency (SIDA) and the UK Department for International Development, to carry out direct government-to-government funding to provincial and district public health systems through the Reproductive, Maternal, Newborn, Adolescent Health and Nutrition Continuum of Care Program, blanketing 6 of 10 provinces. With this partnership alone, over 50% of the Zambian population is covered by projects informed by the SMGL systems approach.^17^ (See the article by Healey et al. from the SMGL supplement.^71^)

### What Were the Main Challenges?

**The initial 1-year time frame.** Frustration was generated when SMGL funding was guaranteed for only 1 year with subsequent support based on achievement of unprecedented reductions in maternal mortality within a highly compressed time frame. At the end of Phase 1 implementation (June 2013) and before results from the Phase 1 endline studies were available (December 2013), host countries and implementing partners were without SMGL funds. Yet they were expected to continue with interventions while a decision on continuation was made. This 6-month period from July to December 2013 was chaotic. Any future systems approach focused on maternal and newborn mortality reduction should commit to a minimum of 5 years of support from the outset.^29^ (See the article by Palaia et al. from the SMGL supplement.^73^)

Future systems approaches focused on maternal and newborn mortality reduction should commit to a minimum of 5 years of support.

**The heavy management burden.** SMGL was a partnership (all U.S. government) within a partnership (countries, a global corporation, nongovernmental organizations, and a professional society). Each partner had a different bottom line, constituency, funding timeline, requirements, and restrictions that all needed to be forged into a dynamic force for change. The positive driver was the ongoing commitment of all partners and stakeholders to dramatically reduce maternal deaths. When the SMGL Leadership Council was recruiting additional countries for SMGL at the end of Phase 1, “management burden” was cited by USAID Mission directors and CDC country office directors as their main concern and rationale for not engaging. A simpler management structure where partnerships provide direct-to-government support with appropriate oversight and ample technical assistance might produce similar results; it might also accelerate country self-sufficiency and increase value for money by decreasing implementing partner overhead charges. At the same time, the diversity of SMGL partners encouraged innovation and enabled access to a wide array of expertise and experience.

**Erratic funding.** Because of the complexity of the partnership and its myriad resource streams, funding to the implementing partners in both countries was profoundly delayed for several periods during Phase 2. These lapses in funding were the result of prolonged U.S. government procurement processes, changes in funding mechanisms, and delays in disbursements from agency headquarters to country offices. If public–private partnerships are increasingly used to advance the goals of U.S. government agencies, streamlining funding for these endeavors will be needed to increase flexibility and responsiveness and to preserve momentum. Smaller amounts of reliable funding are easier to manage than larger tranches of unpredictable financial support.

### What Were Some of the Unexpected Effects?

Having a range of stakeholders participating in SMGL created a think-tank atmosphere that brought together people with varied talents: obstetricians, midwives, nurses, communications specialists, epidemiologists, and district medical and health officers. It also led to collective yearly planning and country budget creation. In many of the routine implementing partner meetings, organizations would share tasks as well as ideas that crossed bureaucratic and competitive barriers. The bold goal of a rapid 50% reduction in maternal mortality fostered a collaborative “all hands on deck” spirit that inspired district leadership and partners alike.

SMGL's insistence on capturing, analyzing, and reporting all maternal deaths resulted in strengthened data gathering and interpretation by district teams. District-level data were presented and critically reviewed by district M&E staff at routine provincial and regional epidemiological meetings. Results were compared within the provinces and among the different project sites, and served as a motivating factor for good performers and as a call for improvement among less successful districts. The heightened appreciation of the need for quality mortality data accelerated the rollout and practice of maternal and perinatal death surveillance and response in both countries. In Zambia, the district commissioner, as the chair of the audit committee, was made responsible for reporting surveillance and response results locally and at the provincial level. This high-level ownership of data was immediately replicated on a national basis and had the effect of positioning maternal mortality not just as a health concern but also as a broader social issue, bringing in other sectors of government and traditional leaders to grapple with and be accountable for preventing maternal mortality.

Better birth planning, involvement of men, and increased community demand for facility deliveries required leaders to raise awareness and address community concerns in order to change cultural norms. Involvement of chiefs and traditional leaders in Zambia and local councils and religious leaders in Uganda created “change champions” who took on these challenges. However, qualitative research by Greeson et al.^74^ identified punitive actions by Zambian village chiefs and headmen, such as fining a husband a goat if he did not provide a sufficient reason for why his wife delivered at home. Researchers suggested that negative unintended consequences are possible by-products of a “big push” endeavor where pressure to succeed is high.^74^ These “disciplinary” actions were not endorsed by SMGL or the MOH, but they do represent a traditional approach by cultural leaders to induce social change in their communities.

### What Are the Main Recommendations Coming Out of the SMGL Experience?

Given the complexity of the SMGL initiative, extracting lessons learned and turning them into a few salient recommendations is challenging. The following points are put forward in support of SMGL's theory of change and organizing principles:

1. Create a culture of zero tolerance for preventable maternal and newborn deaths at all strata of society including parliamentarians and their constituents.
2. Follow key organizing principles by addressing all 3 delays with interventions that are context-specific and time-bound (e.g., setting a 2-hour ‘time-to-service’ limit for complications and focusing on labor, delivery, and 72 hours postpartum).
3. Assess the gaps in the existing maternity care safety net, created by both public and private providers, in the public health catchment area of interest (e.g., district, woreda, county, local government area).
4. Ensure district-level capacity building around planning, execution, and evaluation; consider working in contiguous areas to achieve economies of scale, reduce management burden, and facilitate greater coordination.
5. Support the local health system; work across the district or relevant administrative units to reinforce the system from communities to health centers to hospitals in order to provide equitable lifesaving care and support for mothers and newborns, and by extension, other community members.
6. Sensitize and mobilize community change agents to accelerate normative change but be aware of potential unintended consequences of a “big push” effort.
7. Count, analyze, and report all maternal and perinatal deaths and cause of death.

## CONCLUSIONS

While a 50% reduction in maternal deaths was not achieved during the initiative, the 44% decrease in MMR in Ugandan SMGL-supported facilities and districts, the 38% decrease in Zambian SMGL-supported facilities, and the 41% decrease in Zambian SMGL districts were substantial. There was a marked increase in facility deliveries in both countries and also in population cesarean delivery rates: a 71% increase (5.3% to 9.0%) in Uganda and a 79% increase (2.7% to 4.8%) in Zambia. Perinatal health outcomes were small but significant: the perinatal mortality rate was reduced by 13% in SMGL-supported facilities in Uganda and by 26% in Zambia. The SMGL goal for reduction of newborn deaths (30%) was not achieved in Zambia or Uganda.

Still at question is whether the SMGL health systems strengthening approach to addressing the 3 delays will be adopted or adapted to other country contexts and implemented by MOHs, donors, and multilaterals. Clearly, the level of management burden is high, and partners, especially bilateral donors, are traditionally not structured to be nimble, proactive, or inventive. Yet several global endeavors could benefit from endorsing the SMGL approach. For example, with expansion of the number of Global Financing Facility countries and GFF emphasis on results-based financing, having a ready approach to improving effective coverage (range plus quality) could accelerate GFF impact. Similarly, the district health systems strengthening approach dovetails closely with the objectives and goals of the WHO Quality, Equity, and Dignity initiative.

SMGL was a bold attempt to show that maternal mortality could be reduced significantly in developing countries over a few years of strategic, synergistic programming. It was inspired by the progress achieved by other U.S. government global initiatives that showed how high-level political leadership, focused public attention, evidence-based demand- and supply-side interventions, a broad coalition of stakeholders, and strong M&E could achieve impressive results in a short time. For many, it was an opportunity to change the narrative around the serious problems pregnant women face in the developing world.

Several global endeavors, such as the Global Financing Facility and the WHO Quality, Equity, and Dignity initiative, could benefit from endorsing the SMGL district health systems strengthening approach.

## Supplementary Material

Supplement 1

## Notes

**Peer Reviewed**

**Funding:** Saving Mothers, Giving Life implementation was primarily funded by the Office of the Global AIDS Coordinator, the U.S. Agency for International Development (USAID), Washington, DC, the Centers for Disease Control and Prevention (CDC), Atlanta, Georgia, Merck for Mothers, and Every Mother Counts. The funding agencies had no influence or control over the content of this article.
