# Supplementary material for: Saving Mothers, Giving Life: It Takes a System to Save a Mother
Source: Glob Health Sci Pract. 2019 Mar 11;7(Suppl 1):S6–S26. doi: 10.9745/GHSP-D-18-00427 (PMC6519673; doi:10.9745/GHSP-D-18-00427)
Supplement: Supplement 1 [file GHSP-D-18-00427_index.html]

Supplement to Saving Mothers, Giving Life: It Takes a System to Save a Mother | Global Health: Science and Practice

## Supplemental material

**Files in this Data Supplement:**

- The Savings Mothers, Giving Life Model - Text s01, DOCX
- Saving Mothers, Giving Life Implementing Partners - Text s02, DOCX
- Saving Mothers, Giving Life Routine Quarterly Indicators - Text s03, DOCX
- Saving Mothers, Giving Life Special Studies Results - Text s04, DOCX
- Comparison of SMGL Outcomes With DHS Surveys and UN Maternal Mortality Estimates - Text s05, DOCX
- Strengthening the Safe Motherhood Ecosystem: A Case Study of Private Health Sector Mobilization in Uganda - Text s06, DOCX
