# Supplementary material for: Saving Mothers, Giving Life: It Takes a System to Save a Mother
Source: Glob Health Sci Pract. 2019 Mar 11;7(Suppl 1):S6–S26. doi: 10.9745/GHSP-D-18-00427 (PMC6519673; doi:10.9745/GHSP-D-18-00427)
Supplement: Supplement 1 [file 18-00427-Conlon-Supplement1.docx]

**SUPPLEMENT 1.** The Savings Mothers, Giving Life Model

To accelerate saving lives of mothers and newborns, the Saving Mothers, Giving Life (SMGL) model employs a systems approach focused at the health district level to ensure that every pregnant woman has access to clean and safe delivery services and, in the event of an obstetric complication, lifesaving emergency care within 2 hours. The model serves to strengthen the existing health network (both public and private) within each district to address the three delays: (1) delays in seeking appropriate services, (2) delays in reaching services, and (3) delays in receiving timely and quality care at the facility.

Attention is also focused on the most vulnerable period for mothers and babies—during labor, delivery, and the first 48 hours postpartum. The SMGL approach further integrates maternal and newborn (MNH) services with HIV services (e.g., HIV counseling and testing and prevention of maternal-to-child transmission services) and postpartum family planning. Linkages with other reproductive health services are also strengthened. Based on global best practices with evidence-based MNH and HIV interventions, and on implementation experiences in Uganda and Zambia, this model recommends that each health district strive to ensure the following systems are in place:

- A sufficient number of public and private facilities with appropriate geographical positioning to provide around-the-clock (24 hours a day, 7 days a week) clean and safe delivery services, quality HIV testing, counseling and treatment (for woman, partner, and baby as appropriate), and essential newborn care for all births in the district.
- At a minimum, 5 emergency obstetric and newborn care (EmONC) facilities (public and private) including at least 1 facility that can provide comprehensive emergency obstetric and newborn care (CEmONC), for a population of 500,000 or per 20,000 births, accessible within 2 hours from a delivery site after the development of a severe obstetric or newborn complication, and for postabortion care.
- A sufficient number of skilled birth attendants to provide, on a consistent basis, quality respectful delivery care, diagnosis and stabilization of complications, and if needed, timely referral for EmONC.
- Performance-based EmONC-trained personnel to provide required signal functions at basic emergency obstetric and newborn care (BEmONC) and CEmONC designated facilities. The workforce requirement necessary to eliminate preventable maternal deaths, has been set at 5.9 skilled health providers (doctors, nurses and midwives) per 1000 population.^1^
- Availability and maintenance of necessary infrastructure and equipment; reliable supplies of commodities and drugs to perform the signal functions and provide newborn essential and special care, as well as HIV testing and treatment; and prevention of maternal-to-child transmission services as appropriate to the level of the facility, on a continuous basis.
- Around-the-clock (24 hours a day, 7 days a week), protocol-driven, integrated (public and private) communication and transportation referral system that ensures women with complications reach emergency services within 2 hours. This includes providing, where appropriate, short-term antenatal lodging for women who live more than 2 hours from an EmONC facility.
- A government-owned health management information and data-gathering system that accurately records every birth, obstetric and newborn complication and treatment provided, and birth outcomes at public and private facilities in the district. A timely, no-fault medical death review performed as follow-up for every institutional maternal and neonatal death with cause of death information used for ongoing monitoring and quality improvement.

**REFERENCES**

1. Bustreo F, Say L, Koblinsky M, Pullum TW, Temmerman M, Pablos-Mendez A. Ending preventable maternal deaths: the time is now. *Lancet Global Health*. 2013;1(4):e176–7. [CrossRef](https://doi.org/10.1016/S2214-109X(13)70059-7). [Medline](http://www.ncbi.nlm.nih.gov/pubmed/25104339)
