# Supplementary material for: Saving Mothers, Giving Life: It Takes a System to Save a Mother
Source: Glob Health Sci Pract. 2019 Mar 11;7(Suppl 1):S6–S26. doi: 10.9745/GHSP-D-18-00427 (PMC6519673; doi:10.9745/GHSP-D-18-00427)
Supplement: Supplement 1 [file 18-00427-Conlon-Supplement2.docx]

### SUPPLEMENT 2. Saving Mothers, Giving Life Implementing Partners

| **Government** | **Implementing Partners** |
| --- | --- |
| **Uganda** | |
| Ministry of Health | Baylor College of Medicine Children's Foundation, Uganda |
| Bureau of Statistics | Population Services International |
| Kabarole District Health Office | Management Sciences for Health |
| Kibaale District Health Office | Association of Obstetrics and Gynecologists of Uganda |
| Kamwenge District Health Office | Uganda Pediatric Association |
| Kyenjojo District Health Office | Uganda Society of Anesthesia |
|  | Marie Stopes Uganda |
|  | Ugandan Health Marketing Group |
|  | University Research Company LLC |
|  | Infectious Disease Institute |
| **Zambia** | |
| Ministry of Health | Zambia Center for Applied Health Research and Development |
| Central Statistics Office | Abt Associates |
| Defense Force Medical Services | John Snow Inc. |
| Ministry of Community Development, Mother, and Child Health | Africare |
| Zambia National Blood Transfusion Service (ZNBTS) | Marie Stopes Zambia |
| Kalomo District Health Office | Project Concern International |
| Lundazi District Health Office | RTI International |
| Mansa District Health Office | Elizabeth Glaser Pediatric AIDS Foundation |
| Nyimba District Health Office | Jhpiego |
|  | Churches Health Association of Zambia |
|  | Johns Hopkins University Affiliate |
|  | Boston University |
|  | University of Zambia |
|  | Population Services International |
|  | Centre for Infectious Disease Research in Zambia |
| **Nigeria** | |
| Cross River State Ministry of Health | Association of General and Private Medical Practitioners of Nigeria |
| Cross River State Government | Association of Grassroots Counsellors of Health and Development Nigeria |
| Federal Ministry of Health | Centre for Healthworks, Development and Research Initiative |
| Cross River Primary Health Care Development Agency | ExpandNet |
|  | Greater Hands Foundation |
|  | IntraHealth |
|  | Nigerian Society of Neonatal Medicine, Cross River State Chapter |
|  | Pathfinder International |
|  | Project C.U.R.E. |
|  | Society of Obstetrics and Gynaecology of Nigeria, Cross River State Chapter |
|  | We Care Solar |
