# Supplementary material for: Saving Mothers, Giving Life: It Takes a System to Save a Mother
Source: Glob Health Sci Pract. 2019 Mar 11;7(Suppl 1):S6–S26. doi: 10.9745/GHSP-D-18-00427 (PMC6519673; doi:10.9745/GHSP-D-18-00427)
Supplement: Supplement 1 [file 18-00427-Conlon-Supplement3.docx]

SUPPLEMENT 3. Saving Mothers, Giving Life Routine Quarterly Indicators

1. Number of maternal deaths (in facilities and communities) by cause
2. Number of pre-discharge perinatal (stillbirths and neonatal) deaths
3. Number of direct obstetric complications treated in a health facility by type of complication and type of facility (both emergency obstetric and newborn care [EmONC] and non-EmONC services)
4. Number of women who have had at least 1 antenatal care visit during the first trimester
5. Number of women who had 4 antenatal care visits
6. Number of facilities where the 7 signal functions that constitute basic emergency obstetric and newborn care (BEmONC) services have been performed in the last 3 months
7. Number of facilities where the 9 signal functions that constitute comprehensive emergency obstetric and newborn care (CEmONC) services have been performed in the last 3 months
8. Number of women delivering in a facility, including BEmONC and CEmONC
9. Number of women who stayed in a mother’s shelter prior to delivery in a facility
10. Number of women who delivered in a facility who received pre-discharge postpartum contraception
11. Number of women provided with prophylactic postpartum uterotonic
12. Number of cesarean sections performed in CEmONC facilities
13. Number of newborns who were not breathing at birth and were successfully resuscitated
14. Number of infants breastfed within 1 hour of birth among facility-based births
15. Number of health care workers who successfully completed an in-service EmONC training program
16. Number of maternal and newborn health care providers who received a mentoring, supportive supervision, or technical assistance visit within the last 3 months
17. Number of trained community members reporting to the health center (i.e., village health team, Safe Motherhood Action Group members, volunteers)
18. Number of functioning ambulances by type of vehicle
19. (Uganda) Percentage of vouchers for transportation to a health facility redeemed among all vouches distributed or sold to women
20. (Uganda) Percentage of vouchers for labor and delivery services redeemed among all vouches distributed or sold to women
21. (Uganda) Number of Clean Birth Kits (e.g., Mama Kits) distributed
22. Percentage of individuals from priority populations who completed a standardized HIV prevention intervention, including the specified minimum components, during the reporting period
23. Number of maternity wards refurbished or expanded
24. Number of mothers’ shelters built or renovated
25. Number of staff housing units built or renovated
26. (Zambia) Number of facilities with functioning health groups; (Uganda) Number of communities with functioning health groups
27. (Zambia) Number of communities where pregnant women had access to a functional transportation system or scheme for emergency referral
28. Percentage of pregnant women with known HIV status (including women who were tested for HIV and received their results)
29. Percentage of HIV-positive pregnant women who received antiretroviral therapy to reduce mother-to-child-transmission during pregnancy and delivery
30. Percentage of infants born to HIV-positive women who had an HIV test done within 2 months of birth
31. Percentage of infants born to HIV-positive pregnant women who were started on co-trimoxazole prophylaxis within 2 months of birth
