# Supplementary material for: Saving Mothers, Giving Life: It Takes a System to Save a Mother
Source: Glob Health Sci Pract. 2019 Mar 11;7(Suppl 1):S6–S26. doi: 10.9745/GHSP-D-18-00427 (PMC6519673; doi:10.9745/GHSP-D-18-00427)
Supplement: Supplement 1 [file 18-00427-Conlon-Supplement4.docx]

### SUPPLEMENT 4. Saving Mothers, Giving Life Special Studies Results

**Community Perspectives of a Three-Delays Model Intervention: A Qualitative Evaluation of Saving Mothers, Giving Life in Zambia**^1^

This study was carried out at baseline in 2011 and endline in 2016. Findings from the baseline led to recommendations for the Saving Mothers, Giving Life (SMGL) initiative to sensitize communities about early antenatal care and health facility delivery, and to invest in Zambia’s emergency transportation systems, maternity waiting homes and health facilities, as well as medical supplies, beds, equipment, and training for health workers. Findings from the endline study are covered in the article by Hazemba and colleagues from this supplement.

**Columbia University Implementation Evaluation of Saving Mothers, Giving Life**^2^

A mixed-methods external evaluation of SMGL Phase 1 implementation examined the reach, extent, fidelity, and dynamic effects of the initiative in order to identify best practices and remaining barriers to reducing maternal mortality. The evaluation found that the majority of implementers, national and district stakeholders, and community respondents believed that SMGL had a positive impact on raising awareness of maternal mortality both within and beyond the focus districts. There were also positive spillover effects on the broader health system in the areas of supply chain management, information systems, and health system governance. Women in the community were enthusiastic about SMGL—specifically with the work of the Safe Motherhood Action Groups in Zambia, and the availability of Mama Kits, transportation, and service vouchers in Uganda. Most women who delivered at home reported that they had intended to deliver in facilities, but the sudden onset of labor and lack of transportation to facilities had intervened. The social pressure applied by chiefs to deliver in facilities was generally perceived as positive, although some women who delivered at home reported they felt stigmatized by their communities.

**Travel-Time Mapping in Uganda**^3^

Distance and travel time are key factors for the delay in reaching care. Using road networks, elevation, land-cover, national parks, and water sources, an accessibility analysis was conducted to create travel-time surfaces. Estimations were then modeled to determine the time it would take, by car, to reach the nearest emergency obstetric and newborn care center from any location within the 4 SMGL-supported districts in Western Uganda. Analysis was conducted in AccessMod 5. The analysis was repeated for walking, cycling, and riding a motorcycle. Findings revealed that project efforts over the life span of the SMGL initiative dramatically decreased travel times to emergency obstetric and newborn care centers under all 4 travel modalities. See the article by Schmitz and colleagues from this supplement.

**ELMA Foundation Report on the Newborn Package in Uganda**^4^

Monitoring program interventions and neonatal outcomes using the BABIES (Birthweight by Age-at-Death Boxes for Intervention and Evaluation System) matrix and perinatal death reviews over a 3-year period (2014–2017) revealed several findings. To create demand, 2,134 village health teams visited 31,084 newborns at home during the first week of life and referred 16,908 mothers and their babies for postnatal care. In the 3 targeted districts, 3,240 radio spots were run, and 99 talk shows and 75 dialogue meetings were held. To increase the proportion of facilities meeting newborn care standards, maternity wings were renovated and equipped for special care of the sick and premature newborns. This increased the number of Special Care Neonatal Units from 3 in 2014 to 8 in 2016. Additional medical commodity procurements and facility upgrades resulted in 85% of health facilities meeting Uganda MOH newborn care standards.

### Investments to Accelerate Reduction in Maternal Mortality: Findings from Expenditure Studies in Uganda and Zambia for the Saving Mothers, Giving Life Partnership^5^

The study measured additional expenditures made by SMGL during Phase 0 and Phase 1 beyond ministry of health inputs that led to a 35% reduction in the maternal mortality ratio in facilities in SMGL-supported districts in Uganda and Zambia. SMGL invested US$18.64 million in the 8 learning districts; expenditures varied widely by district, from US$0.83 million to US$4.16 million depending on the unique gaps and context determined by the health facility assessments at baseline. Capital expenditures included hiring staff; training in safe delivery and emergency obstetric care; procuring emergency transportation, equipment, and furniture; and renovation of health facilities and maternity waiting homes. Recurring expenditures covered medical supplies, mentoring, and health personnel salaries. Demand generation expenditures covered training, equipping, and incentivizing community volunteers, and community mobilization activities. Expenditures for systems support included program oversight, supervision, monitoring and evaluation, and systems strengthening.

Projecting for the number of births for the subsequent 5 years, it was estimated that capital investments would add US$6.93 in Uganda and US$12.74 in Zambia per improved birth. Conclusions included that “costs for program support and supervision will likely decrease dramatically as ownership of SMGL activities is transitioned to host governments and the model is scaled-up because fewer partner-funded staff will be required to support program implementation” (<http://www.savingmothersgivinglife.org/docs/SMGL_Executive_Summary.pdf>).

### Uganda Qualitative Comparative Analysis Cost-Effectiveness Study^6^

A qualitative comparative analysis approach with cost modeling was used to conduct a cost-effectiveness analysis of SMGL Phase 1 results. Different bundles of interventions (pathways) were compared by cost and effectiveness in regard to each bundle’s capacity to achieve the reported 30% reduction in the Ugandan community maternal mortality ratio at the end of Phase 1. The study found that the less expensive and most effective bundle of interventions, expressed in cost per improved birth, followed the 3 delays theory of change model: village health teams (demand and self-care); motorcycle (*boda boda*) transport vouchers (timely access to care); mentoring of existing staff (quality); and access to medicine (health systems strengthening). The added cost of this package for year 1, including program management costs of US$18 per improved birth, was US$46 per improved birth. Authors note that costs would likely lower for year 2 and beyond as efficiency improved and capital costs decreased.

**The Costs and Cost-Effectiveness of a District Strengthening Strategy to Mitigate the 3 Delays to Quality Maternal Health Care: results from Uganda and Zambia**^7^

To measure effectiveness, data from baseline and endline censuses in Zambia^51^ and Reproductive Age Mortality Surveys in Uganda were analyzed. For costs, data from health facilities, ministerial and health offices, and implementing partners for the year 2016 were collected in 2 of the 4 SMGL learning districts in each country and in 3 comparison districts (2 in Zambia, 1 in Uganda). In Uganda, the incremental cost per SMGL improved delivery was US$38, while in Zambia it was US$95 per improved birth. For the SMGL-supported districts included in this study, scale-up of maternal and newborn health activities is associated with 164 maternal and perinatal deaths averted in Uganda and 121 deaths averted in Zambia when comparing 2016 to 2012. In Uganda, the cost per life-year gained was US$177 and in Zambia it was US$206. Authors conclude that the district health systems strengthening approach addressing the 3 delays represents a cost-effective intervention. See the article by Johns and colleagues in this supplement.

**REFERENCES**

1. Ngoma-Hazemba A, Soud F, Hamomba L, Silumbwe A, Munakampe MN, Spigel L; Saving Mothers, Giving Life Working Group. Community perceptions of a 3-delays model intervention: a qualitative evaluation of Saving Mothers, Giving Life in Zambia. *Glob Health Sci Pract*. 2019;7(suppl 1). [CrossRef](https://doi.org/10.9745/GHSP-D-18-00287)
2. Kruk M, Galea S, Grepin K, Rabkin M. *External Evaluation of Saving Mothers Giving Life: Final Report.* New York: Columbia University Mailman School of Public Health; 2013. <http://pdf.usaid.gov/pdf_docs/pbaaf149.pdf>. Accessed June 18, 2018.
3. Schmitz MM, Serbanescu F, Kamara V, et al; Saving Mothers, Giving Life Working Group. Did the Saving Mothers, Giving Life initiative expand timely access to lifesaving care in Uganda? A spatial district-level analysis of travel time to emergency obstetric and newborn care. *Glob Health Sci Pract*. 2019;7(suppl 1). [CrossRef](https://doi.org/10.9745/GHSP-D-18-00366)
4. Baylor College of Medicine Children’s Foundation Uganda (Baylor-Uganda). *Interim Narrative Report, Grant # 14-F0017, August 2017*. Kampala: Baylor-Uganda; 2017. <https://www.baylor-uganda.org/download/annual_reports/Final-Baylor-Narrative-Report-Aug-17-2017.pdf>. Accessed December 14, 2018.
5. Futures Group, Health Policy Project. *Investments to Accelerate Reductions in Maternal Mortality: Findings from Expenditure Studies in Uganda and Zambia for the Saving Mothers, Giving Life Partnership*. Washington, DC: Futures Group; 2014. <http://www.savingmothersgivinglife.org/docs/USAID-SMGL-Expenditure-Study-Executive-Summary.pdf>. Accessed July 11, 2018.
6. Saving Mothers, Giving Life Phase 1 in Uganda: cost effectiveness analysis. USAID/Uganda Monitoring, Evaluation and Learning Program. AID-617-c-13-00007. SoCha.IIc. 2016.
7. Johns B, Hangoma P, Atuyambe L, et al; Saving Mothers, Giving Life Working Group. The costs and cost-effectiveness of a district-strengthening strategy to mitigate the 3 delays to quality maternal health care: results from Uganda and Zambia. *Glob Health Sci Pract*. 2019;7(suppl 1). [CrossRef](https://doi.org/10.9745/GHSP-D-18-00429)
