# Supplementary material for: Saving Mothers, Giving Life: It Takes a System to Save a Mother
Source: Glob Health Sci Pract. 2019 Mar 11;7(Suppl 1):S6–S26. doi: 10.9745/GHSP-D-18-00427 (PMC6519673; doi:10.9745/GHSP-D-18-00427)
Supplement: Supplement 1 [file 18-00427-Conlon-Supplement5.docx]

### SUPPLEMENT 5. Comparison of SMGL Outcomes With DHS Surveys and UN Maternal Mortality Estimates

The authors conducted several secondary analyses comparing the results of Saving Mothers, Giving Life (SMGL) studies and the results of Demographic and Health Surveys (DHS) or United Nations (UN) maternal mortality estimates.

### Comparing SMGL Results With Findings of DHS Surveys in Uganda

Maternal mortality ratios (MMR), facility delivery coverage, and percentage of births delivered by caesarean section were calculated by the Reproductive Age Mortality Survey (RAMOS) for SMGL districts as described in the article. For this secondary analysis we calculated the standard error for MMR using a Poisson distribution. For facility delivery and C-section, standard errors were based on the normal approximation to the binomial distribution, with a standard error = (p*(1-p)/n)^1/2^.

We calculated the average annual rate of reduction (ARR) in the SMGL Phase 1 districts of Uganda and Zambia as 100*(log(MMRendline/MMRbaseline)/5), consistent with other papers in this series and World Health Organization (WHO) methodology.^[[1]](#endnote-1)^

For MMR in Uganda, the RAMOS estimates were compared to the Uganda DHS from 2006, 2011, and 2016. MMR in DHS was estimated for the entire country, so the entire country (and standard errors) was used as the control. Additionally, in 2017 DHS changed their definition of maternal mortality to align with the WHO and the RAMOS definitions.^[[2]](#endnote-2)^ Therefore, we adjusted the values for MMR for the 2006 and 2011 surveys by using the ratio published in the 2016 DHS (which calculated MMR using both the new and old method), to align all values with the same methodology.

Because the time period for data collection for the RAMOS and DHS aligned almost exactly, both spanning five years, we were able to compare the two datasets.

We tested whether MMR declined significantly faster (*P* < .05) in SMGL Phase 1 districts compared with Uganda as a whole using the difference-in-difference (DID) method, as was done in a separate analysis comparing specific maternal and child health interventions for SMGL Phase 1 districts to nearby districts:^[[3]](#endnote-3)^

Z = (MMR_RAMOS_2011_ - MMR_RAMOS_2016_) - (MMR_DHS_2011_ – MMR_DHS_2016_)

$${SE}_{DID}=\sqrt{{SE}_{RAMOS_{2016}}^{2}+ {SE}_{RAMOS_{2011}}^{2}+ {SE}_{DHS_{2016}}^{2}+ {SE}_{DHS_{2011}}^{2}}$$

We also calculated the national ARR in Uganda using the DHS from 2011 and 2016 as (100*log(MMR_2016_/MMR_2011_)/5).

### Comparing SMGL Results With UN Maternal Mortality Estimates in Zambia

The timing of the recent DHSs in Zambia (2013 and 2018) did not allow us to use the DHS for a comparison analysis. For the Zambia national comparison, we analyzed the UN maternal mortality estimates carried out by the UN Maternal Mortality Estimation Inter-Agency Group (MMEIG).^1^ To calculate a national ARR for MMR, we simply used the most recent estimates from the MMEIG. The last estimates were published for 2015. So, the ARR calculation for Zambia was (100*log(MMR_2015_/MMR_2011_)/4).

**Results**

MMR declined significantly faster in SMGL Phase 1 districts than in the rest of Uganda (Figure A). The ARR for maternal mortality seen in the Uganda SMGL districts versus the national DHS ARR was 11.5% and 3.5%, respectively (Difference In Difference: *P* = .017). The findings for Zambia are similar. Between 2011 and 2015, the years for available MMEIG estimates, the ARR in SMGL LDs in Zambia was 10.5% compared with a national ARR of 2.8%. These findings argue against the SMGL health outcomes being due solely to a secular trend. These improvements were also associated with significantly faster improvements in Uganda in both facility delivery and percent of births delivered by C-section (Figures B and C). Note also that approximately 70% of the improvement came in the first year of the program.

**Figure A. Comparing Maternal Mortality in Uganda SMGL Districts with the DHS, 2006-2016.**

**Figure B. Comparing Facility Deliveries in Uganda SMGL Districts with the DHS, 2006-2016.**

**Figure C. Comparing C-section rates in Uganda SMGL Districts with the DHS, 2006-2016.**

The rate of reduction in maternal mortality in Uganda and Zambia SMGL-supported districts during the program period is greater than the national rate of reduction over the same period. The association of the maternal mortality reduction in Uganda SMGL areas with increased facility delivery and C-section rates suggest the contribution of SMGL to increase demand for, access to, and quality of maternal health services in the SMGL areas. These findings align with a separate analysis that was conducted by the DHS program for Uganda, which found that C-section rates increased more rapidly in SMGL districts than others.^3^ In that same analysis, as an indicator that quality of maternal services was increasing, the frequency of blood pressure measurement during antenatal care visits improved significantly more in SMGL districts compared to other districts.

Most of the improvement in maternal mortality in Uganda appears to have come in the first phase coincident with the most intensive level of resources and urgency. However, the maternal mortality ratio continued to decline after Phase 1 at a slower rate. This suggests that gains can at least be sustained by the level of resources and efforts provided after Phase 1, a level that countries are more likely to be able to maintain over a longer period.

1. **References**

   . World Health Organization (WHO). *Trends in maternal mortality: 1990 to 2015: estimates by WHO, UNICEF, UNFPA, World Bank Group and the United Nations Population Division.* Geneva: WHO; 2015. [↑](#endnote-ref-1)
2. . The DHS Program: Demographic and Health Surveys. The DHS Program Blog. <https://blog.dhsprogram.com/mmr-prmr/>. Accessed July 11, 2018.

   [↑](#endnote-ref-2)
3. . Mallick L, Dontamsetti T, Pullum T, Fleuret J. *Using the Uganda Demographic and Health Surveys from 2011 and 2016 to Assess Changes in Saving Mothers, Giving Life Intervention Districts.* Rockville, MD: ICF; 2018. <https://dhsprogram.com/pubs/pdf/WP142/WP142.pdf>. [↑](#endnote-ref-3)
