# Supplementary material for: Saving Mothers, Giving Life: It Takes a System to Save a Mother
Source: Glob Health Sci Pract. 2019 Mar 11;7(Suppl 1):S6–S26. doi: 10.9745/GHSP-D-18-00427 (PMC6519673; doi:10.9745/GHSP-D-18-00427)
Supplement: Supplement 1 [file 18-00427-Conlon-Supplement6.docx]

**SUPPLEMENT 6.** Strengthening the Safe Motherhood Ecosystem: A Case Study of Private Health Sector Mobilization in Uganda

Preventable maternal deaths are the result of multiple, intersecting failures of an entire health care system to provide a woman the care she needs, when she needs it. In Uganda, Saving Mothers, Giving Life (SMGL) sought to unlock the potential of the private sector to address the three delays and improve access to and quality of maternity care. Standardized and quality-assured maternal health services (antenatal, labor and delivery, postnatal and postpartum family planning) were introduced into the *ProFam* network of family planning providers, led by the Program for Accessible Health Communication and Education (PACE)*.* Private providers, midwives managing at least 8-10 births per month, also received training to bolster their business models and the referral linkages between their clinics and public facilities.

By the end of the initiative, there were marked improvements in the three quality indicators measured across the 17 private facilities supported by PACE in the four SMGL learning districts:

- Total number of deliveries rose by 21%, from 668 (2014) to 809 (2016)
- Frequency of blood pressure checks upon admission rose from 78% (2015) to 100% (2016)
- Administration of a uterotonic immediately following delivery increased from 79% (2015) to 100% (2016)

Lessons learned from the Uganda PACE experience include:

1. The importance of identifying and engaging private providers with the capacity and caseload to take on quality labor and delivery care. Lower-volume private facilities can offer high quality antenatal and postnatal care through targeted skill-building efforts that focus on identifying higher-risk pregnancies for timely and appropriate referrals.
2. Nurses and midwives play a critical role in triaging cases expeditiously within a coordinated system. Strengthening the capacity of private facilities to manage a greater share of uncomplicated deliveries and respond more effectively to simple complications can help decongest public health centers.
3. The need to become sustainable *as a business* drives innovation in care, as private providers seek new ways to serve their patients and gain a competitive edge. Advocacy efforts to ensure that health insurance schemes include private maternity care and set minimum quality requirements for provider empanelment are ways to use market dynamics to improve access to quality.
4. To achieve universal health care coverage, there is a need for stronger stewardship of private care through new approaches to certification and accreditation. Assessing the content of care is difficult in settings with relatively few deliveries because of the limited opportunities to directly observe provider skills. PACE assessed private provider competencies by conducting simulations and emergency obstetric drills, developing case studies on managing complications, and by reviewing documentation.

Strengthening the safe motherhood ecosystem requires meaningful engagement with local private providers to build their capacity to deliver quality, timely and appropriate care. It also requires engaging with drug shops, community health workers, transportation operators and other private actors who play critical roles addressing the three delays. It will be important to apply these lessons from Uganda in the design, implementation, and evaluation of future efforts to integrate local private sector in total health system strengthening approaches. With up to 40% of women in LMICs receiving delivery care from local private providers, too many lives are depending on it.

Authors:

Angela L. Coral, Rabin Martin; Hanna Baldwin, Population Services International Uganda/Program for Accessible Health Communication and Education; Dorothy Balaba, Population Services International Uganda/Program for Accessible Health Communication and Education; Mary-Ann Etiebet, MSD for Mothers

**References**

1. Campbell O, Benova L, Macleod D, et al. Family planning, antenatal and delivery care: cross-sectional survey evidence on levels of coverage and inequalities by public and private sector in 57 low- and middle-income countries. *Trop Med Int Heal*. 2016. doi:10.1111/tmi.12681.
